# Evaluation of the Dietary Supplementation of a Formulation Containing Ascorbic Acid and a Solid Dispersion of Curcumin with Boric Acid against *Salmonella* Enteritidis and Necrotic Enteritis in Broiler Chickens

**DOI:** 10.3390/ani9040184

**Published:** 2019-04-22

**Authors:** Daniel Hernandez-Patlan, Bruno Solís-Cruz, Karine Patrin Pontin, Juan D. Latorre, Mikayla F. A. Baxter, Xochitl Hernandez-Velasco, Ruben Merino-Guzman, Abraham Méndez-Albores, Billy M. Hargis, Raquel Lopez-Arellano, Guillermo Tellez-Isaias

**Affiliations:** 1 Laboratorio 5: LEDEFAR, Unidad de Investigacion Multidisciplinaria, Facultad de Estudios Superiores Cuautitlan, Universidad Nacional Autonoma de México, Cuautitlan Izcalli 54714, Mexico; danielpatlan@comunidad.unam.mx (D.H.-P.); bruno_sc@comunidad.unam.mx (B.S.-C.); rlajjd@yahoo.com.mx (R.L.-A.); 2 Departamento de Medicina Veterinária Preventiva, Centro de Diagnóstico e Pesquisa em Patologia Aviária, Universidade Federal do Rio Grande do Sul, Porto Alegre RS 97105-900, Brazil; pontin.karine@gmail.com; 3 Department of Poultry Science, University of Arkansas, Fayetteville, AR 72704, USA; juandlatorre@gmail.com (J.D.L.); mb022@email.uark.edu (M.F.A.B.); bhargis@uark.edu (B.M.H.); 4 Departamento de Medicina y Zootecnia de Aves, Facultad de Medicina Veterinaria y Zootecnia, Universidad Nacional Autónoma de México, Mexico City 04510, Mexico; xochitl_h@yahoo.com (X.H.-V.); onirem@unam.mx (R.M.-G.); 5 Laboratorio 14: Alimentos, Micotoxinas y Micotoxicosis, Unidad de Investigacion Multidisciplinaria, Facultad de Estudios Superiores Cuautitlan, Universidad Nacional Autonoma de Mexico, Cuautitlan Izcalli 54714, Mexico; albores@unam.mx

**Keywords:** chickens, ascorbic acid, curcumin, boric acid, necrotic enteritis, *Salmonella* Enteritidis

## Abstract

**Simple Summary:**

Prophylactic or therapeutic administration of a 0.1% mixture containing ascorbic acid (AA) and a solid dispersion of curcumin (CUR) with polyvinylpyrrolidone (PVP) and boric acid (BA) (AA-CUR/PVP-BA) significantly reduced the concentration of *Salmonella* Enteritidis in broiler chickens and had a positive effect in slightly diminishing the negative impact of necrotic enteritis (NE).

**Abstract:**

Two experiments were conducted to evaluate the effect of the prophylactic or therapeutic administration of a 0.1% mixture containing ascorbic acid and a solid dispersion of curcumin with polyvinylpyrrolidone and boric acid (AA-CUR/PVP-BA) against *Salmonella* Enteritidis (*S*. Enteritidis) in broiler chickens. A third experiment was conducted to evaluate the impact of the dietary administration of 0.1% AA-CUR/PVP-BA in a necrotic enteritis (NE) model in broiler chickens. The prophylactic administration of 0.1% AA-CUR/PVP-BA significantly decreased *S*. Enteritidis colonization in cecal tonsils (CT) when compared to the positive control group (PC, *p* < 0.05). The therapeutic administration of 0.1% AA-CUR/PVP-BA significantly reduced the concentration of *S*. Enteritidis by 2.05 and 2.71 log in crop and CT, respectively, when compared with the PC on day 10 post-*S*. Enteritidis challenge. Furthermore, the serum FITC-d concentration and total intestinal IgA levels were also significantly lower in chickens that received 0.1% AA-CUR/PVP-BA. Contrary, the PC group showed significantly higher total intestinal IgA levels compared to the negative control or AA-CUR/PVP-BA groups in the NE model. However, 0.1% AA-CUR/PVP-BA showed a better effect in reducing the concentration of *S*. Enteritidis when compared to the NE model. Further studies with higher concentration of AA-CUR/PVP-BA into the feed to extend these preliminary results are currently being evaluated.

## 1. Introduction

In the poultry industry, enteric bacterial pathogens pose a threat to intestinal health and can contribute to the transmission of zoonotic diseases [1,2], increased mortality in poultry flocks, reduced feed efficiency, decreased rate of body weight gain and, therefore, increase in total production costs [3,4]. *Salmonella* infection and necrotic enteritis (NE) produced by *Clostridium perfringens* (CP) are two significant bacterial diseases in poultry [5,6]. Each year, millions of foodborne salmonellosis cases are reported, resulting in an estimated 155,000 deaths [6]. The most common route of transmission from animals to humans is through contaminated food such as meat, eggs and meat-based products [7,8]. It has also been reported that the presence of salmonellosis has caused significant economic losses in poultry production due to the reduction in overall performance and high mortality in affected flocks [4,9]. Another economically significant disease affecting chicken production is NE induced by CP and occurs in two forms. In its acute clinical form, NE can cause significant flock mortality [10,11,12] for several days, whereas the subclinical and chronic form can significantly impair performance [13,14]. The economic impact of NE on the worldwide poultry industry was estimated at over five to six billion dollars per year [15]. Thus, controlling enteric bacterial disease in poultry is essential to maintain efficient production and improve food safety [2].

Restrictions on the use of antimicrobials at sub-therapeutic doses in animal production [16] have pressured the poultry industry to look for alternatives to reduce the problems of bacterial resistance and also continue to provide performance benefits, eliminating foodborne pathogens as *Salmonella*, and reducing the NE incidence. Some of these alternatives include probiotics (yeasts or bacteria), plant derivatives such as essential oils or extracts, organic acids, enzymes and lysozymes [2,17,18].

A recent in vitro study published by our laboratory demonstrated the capability of 1% ascorbic acid (AA) to significantly reduce the concentration of *Salmonella* Enteritidis (*S*. Enteritidis) in the compartment that simulates the crop, derived from its acidification capacity, but not in the intestinal compartment since it degrades as the pH increases [19]. Another study showed that broiler chickens supplemented with 0.1% of a solid dispersion of curcumin (CUR) with polyvinylpyrrolidone (PVP) and boric acid (BA, CUR/PVP-BA) resulted in a lower *S*. Enteritidis recovery in both crop and cecal tonsils (CT) because of a possible synergistic effect between them [20]. Therefore, the purposes of the present study were to evaluate the effect of the prophylactic or therapeutic administration of a 0.1% mixture containing AA and a solid dispersion of CUR with PVP and BA (AA-CUR/PVP-BA) in broiler chickens infected with *S*. Enteritidis, as well as the impact of the dietary administration of 0.1% AA-CUR/PVP-BA in broilers using a laboratory NE challenge model.

## 2. Materials and Methods

### 2.1. Preparation of Treatments and Diets

The mixture containing AA and CUR/PVP-BA (AA-CUR/PVP-BA) were prepared in two steps. The first step involved the preparation of the solid dispersion of CUR/PVP-BA (1:1 ratio) as previously described [19]. Subsequently, a mixture of 90% of AA and 10% of microcrystalline cellulose (MCC) pH 10.2 was granulated, dried, sieved and finally associated with the solid dispersion of CUR/PVP-BA. The proportion of each component was 33.3% (1:1:1, AA:CUR/PVP:BA) and the particle size obtained was around 700 µm. The AA-CUR/PVP-BA mixture was mixed into the feed for 15 min using a rotary mixer to obtain the experimental diet with a final concentration of 0.1% (1 g/kg of feed). The starter feed used in this study was formulated to approximate the nutritional requirements for broiler chickens as recommended by the National Research Council [21], and adjusted to the breeder’s recommendations [22]. No antibiotics, coccidiostats or enzymes were added to the feed (Table 1). All animal handling procedures complied with the Institutional Animal Care and Use Committee (IACUC) at the University of Arkansas, Fayetteville (protocol #15006).

### 2.2. Salmonella Strain and Culture Conditions

A primary poultry isolate of *Salmonella enterica* serovar Enteritidis (*S*. Enteritidis) bacteriophage type 13A, was obtained from the USDA National Veterinary Services Laboratory (Ames, IA, USA). This strain is resistant to 25 µg/mL of novobiocin (NO, catalog no. N-1628, Sigma, St. Louis, MO, USA) and was selected due to its resistance to 20 µg/mL of nalidixic acid (NA, catalog no. N-4382, Sigma, St. Louis, MO, USA) in our laboratory. In the present study, 100 µL of *S*. Enteritidis from a frozen aliquot were added to 10 mL of tryptic soy broth (TSB, Catalog No. 22092, Sigma, St. Louis, MO, USA) and incubated at 37 °C for 8 h, and passed three times every 8 h to ensure that all bacteria were in log phase as previously described [23]. Post-incubation, bacterial cells were washed three times with sterile 0.9% saline by centrifugation at 1864× *g* for 10 min, reconstituted in saline, quantified by densitometry with a spectrophotometer (Spectronic 20DC, Spectronic Instruments Thermo Scientific, Rochester, NY, USA) and finally diluted to an approximate concentration of 4 × 10^4^ cfu/mL and 4 × 10^7^ cfu/mL. Concentrations of *S*. Enteritidis were further verified by serial dilutions and plated on brilliant green agar (BGA, Catalog No. 70134, Sigma, St. Louis, MO, USA) with NO and NA for enumeration of actual cfu used in the experiment.

### 2.3. Experiment 1

Two independent trials were conducted to evaluate the prophylactic administration of 0.1% AA-CUR/PVP-BA in reducing the incidence of *S*. Enteritidis in broiler chickens. In each trial, 30 day-of-hatch male Cobb-Vantress broiler chickens (Fayetteville, AR, USA) were randomly allocated to one of two groups (*n* = 15 chickens): (1) Group challenged with *S*. Enteritidis (positive control group, PC) and (2) 0.1% (w/w) AA-CUR/PVP-BA into feed and challenged with *S*. Enteritidis (AA-CUR/PVP-BA group). Chicks were housed in brooder battery cages, provided with their respective diet and water ad libitum, and maintained at an age-appropriate temperature during the seven days of the experiment. On day six of age, all chicks were orally challenged with 1 × 10^7^ cfu of *S*. Enteritidis per bird. Chicks were euthanized by CO_2_ inhalation 24 h post-*S*. Enteritidis challenge (Day 7), and samples of crop and CT were taken for *S*. Enteritidis recovery. 

### 2.4. Experiment 2

The purpose of experiment 2 was to evaluate the effectiveness of the therapeutic administration of 0.1% AA-CUR/PVP-BA in broiler chickens infected with *S*. Enteritidis during three and 10 days of treatment. For this, an experiment with 60 one-day-old male Cobb-Vantress broiler chickens (Fayetteville, AR, USA) were challenged with 1 × 10^4^ *S*. Enteritidis cfu per bird and randomly allocated to one of two groups (*n* = 30 chickens): (1) Positive control group (PC); (2) 0.1% (w/w) AA-CUR/PVP-BA into the feed (AA-CUR/PVP-BA group). Chicks were housed in brooder battery cages, provided with their respective diet and water ad libitum, and maintained at an age-appropriate temperature during the 10 days of the experiment. On days three and 10 post-*S*. Enteritidis challenge, 15 chicks from each group were euthanized by CO_2_ inhalation, and the crop and CT from 12 birds per group were aseptically collected to evaluate *S*. Enteritidis recovery. Blood samples were collected from the femoral vein and centrifuged (1000× *g* for 15 min) to separate the serum for the determination of fluorescein isothiocyanate-dextran (FITC-d) concentration on day 10 post-*S*. Enteritidis challenge. The concentration of FITC-d administered was calculated based on group body weight at day 9 post-*S*. Enteritidis challenge. Furthermore, intestinal samples for total intestinal IgA levels were also collected.

### 2.5. Salmonella Recovery

In experiment 1 and 2, the crop and CT were homogenized and diluted with saline (1:4 w/v), and 10-fold dilutions were plated on BGA with NO and NA, incubated at 37 °C for 24 h to enumerate total *S*. Enteritidis colony forming units. Subsequently, the crop and CT samples were enriched in 2 × concentrated tetrathionate enrichment broth and further incubated at 37 °C for 24 h. Enrichment samples were streaked onto Xylose Lysine Tergitol-4 (XLT-4, Catalog No. 223410, BD DifcoTM) selective media for confirmation of *Salmonella* presence.

### 2.6. Experiment 3

This experiment was conducted to evaluate the impact of the dietary administration of 0.1% AA-CUR/PVP-BA on growth performance, intestinal barrier integrity and ileum lesions in broiler chickens using a laboratory necrotic enteritis (NE) challenge model. One hundred and twenty day-of-hatch male Cobb-Vantress broiler chickens (Fayetteville, AR, USA) were randomly assigned to three different groups of four replicates each with ten broiler chickens (*n* = 40/group): (1) Non-challenged control (negative control group, NC), (2) challenged control (positive control group, PC) and, (3) challenge control + 0.1% AA-CUR/PVP-BA into the feed (AA-CUR/PVP-BA group). All chicks were raised in floor pens (300 cm × 150 cm) for 21 days, provided with their respective diet and water ad libitum, and maintained at an age-appropriate temperature protocol during the experiment. On day 21, broiler chicks were euthanized by CO_2_ inhalation, and the right half of the liver from 12 broiler chickens was aseptically collected in sterile sample bags (Nasco, Fort Atkinson, WI, USA) to evaluate bacterial translocation. Additionally, blood samples were collected from the femoral vein and centrifuged (1000× *g* for 15 min) to separate the serum for FITC-d estimation. The concentration of FITC-d administered was calculated based on group body weight at 20-day-old. Likewise, intestinal samples for the measurement of total intestinal IgA levels were also collected. Ileum NE lesion scores (ILS, *n* = 25 broiler chickens/group) were evaluated as recommended by Hofacre [24]: 0 = No lesions; 1 = thin-walled and friable intestines; 2 = focal necrosis, gas production and ulceration; 3 = extensive necrosis, hemorrhage and gas-filled intestines; and 4 = generalized necrosis typical of field case, marked hemorrhage. Finally, body weight (BW) and body weight gain (BWG) were evaluated on a weekly basis. Feed intake (FI) and feed conversion ratio (FCR) were obtained at 21-d of age.

### 2.7. NE Model: Challenge or Ganisms

NE was induced in the broiler chickens as previously described [25,26] with slight modifications. Briefly, day-old broiler chickens were challenged with a concentration of 1 × 10^8^ cfu of *Salmonella* Typhimurium (ST) per bird by oral gavage. This organism was isolated from poultry and obtained from the USDA National Veterinary Services Laboratory (Ames, IA, USA). The isolate was resistant to novobiocin (25 µg/mL of NO, catalog no. N-1628, Sigma) and was selected for resistance to nalidixic acid (20 µg/mL of NA, catalog no. N-4382, Sigma) in our laboratory. ST culture was performed in the same way as described above for *S*. Enteritidis. However, ST suspension was diluted to an approximate concentration of 4 × 10^8^ cfu/mL. The concentration of ST was further verified by serial dilution and plated on brilliant green agar (BGA, Catalog no. 70134, Sigma) with NO and NA for enumeration of actual cfu used in the experiment. Subsequently, at day 13 of age, broiler chickens were challenged with a dose of 2 × 10^4^ sporulated oocysts of *Eimeria maxima* (EM) per bird by oral gavage. Oocysts were propagated in vivo, according to previously published methods [27,28] and a preliminary dose titration study was carried out, offset by one week, to determine the EM challenge selection for the present study. At day 18 of age, chickens were challenged with a concentration of 1 × 10^9^ cfu of a mixture of two *Clostridium perfringens* (CP) isolates per bird by oral gavage. Dr. Jack McReynolds (USDA-ARS, College Station, TX, USA) kindly donated the first strain of CP previously described in an NE challenge model [29]. The second strain was isolated from a separate *Eimeria* challenge experiment in our laboratory with an inadvertent resulting NE (four weeks of age). Then, a single aliquot of each isolate was individually amplified in TSB with thioglycolate (Catalog no. 212081, Becton Dickinson, Sparks, MD, USA) overnight and subsequently mixed. Plating 10-fold dilutions confirmed the concentration of CP on phenylethyl alcohol agar plates (PEA, Becton Dickinson, Sparks, MD, USA) with 5% sheep blood (Remel, Lenexa, KS, USA).

### 2.8. Liver Bacterial Translocation (BT)

Briefly, liver samples were homogenized, weighed and diluted 1:4 w/v with sterile 0.9% saline enriched with sodium thioglycolate. Then, 10-fold dilutions were plated on tryptic soy agar (TSA, catalog no. 211822, Becton Dickinson, Sparks, MD, USA) with thioglycolate for anaerobic bacteria (AB) recovery. Plates were then incubated anaerobically at 37 °C for 24 h to enumerate entire AB colony forming units per g of tissue.

### 2.9. Serum Determination of FITC-d Leakage

FITC-d (MW 3–5 kDa; Sigma-Aldrich Co., St. Louis, MO, USA) was used as a marker of paracellular transport and mucosal barrier dysfunction [30,31]. One hour before the chicks were euthanized by CO_2_ inhalation, 12 or 20 broiler chickens from each group were given an oral gavage dose of FITC-d (8.32  mg/kg of body weight), and three or five broiler chickens per group were used as controls for the experiment 2 or 3, respectively. The concentrations of FITC-d from diluted sera were measured fluorometrically at an excitation wavelength of 485 nm and an emission wavelength of 528 nm (Synergy HT, Multi-mode microplate reader, BioTek Instruments, Inc., VT, USA) [32].

### 2.10. Total Intestinal Immunoglobulin A (Iga) Levels

Total IgA levels in experiments 2 and 3 were determined in 12 gut rinse samples each as previously described [33]. An intestinal section of 5 cm from the Meckel’s diverticulum to the ileocecal junction was taken and rinsed three times with 5 mL of 0.9% saline; then the rinse was collected in a tube and centrifuged at 1864× *g* at 4 °C for 10 min. The supernatant was poured into a 96-microwell plate and stored at −20 °C until tested. A commercial indirect ELISA set was used to quantify IgA according to the manufacturer’s instructions (Catalog No. E30-103, Bethyl Laboratories Inc., Montgomery, TX 77356, USA). 96-well plates (Catalog No. 439454, Nunc MaxiSorp, Thermo Fisher Scientific, Rochester, NY, USA) were used, and samples diluted 1:100 were measured at 450 nm using an ELISA plate reader (Synergy HT, multi-mode microplate reader, BioTek Instruments, Inc., Winooski, VT, USA). Total intestinal IgA levels obtained were multiplied by the dilution factor (100) to determine the amount of chicken IgA in the undiluted samples.

### 2.11. Data and Statistical Analysis

Data from *S*. Enteritidis and AB counts (Log cfu/g), BW, BWG, FI, FCR, total IgA levels, serum FITC-d concentration and ileum NE lesion score were subjected to an analysis of variance (ANOVA) as a completely randomized design, using the general linear models procedure of Statistical Analysis System (SAS) [34]. The experimental unit for each variable is reported in each table respectively. Significant differences among the means were determined by Duncan’s multiple range test at *p* < 0.05. Enrichment data were expressed as positive/total chickens (%), and the recovery percentage of AB and *S*. Enteritidis were compared using the chi-squared test of independence [35], testing all possible combinations to determine the significance (*p* < 0.05).

## 3. Results

The results of the prophylactic administration of 0.1% AA-CUR/PVP-BA on *S*. Enteritidis colonization in the crop and CT of broiler chickens in trials 1 and 2 (Exp 1) are summarized in Table 2. Although there was no reduction in *S*. Enteritidis colonization in the crop of chickens treated with 0.1% AA-CUR/PVP-BA into the feed in both trials, in CT, the concentration of *S*. Enteritidis significantly decreased by more than 1.6 log in comparison with the positive control (PC, *p* < 0.05). Furthermore, chickens receiving 0.1% of AA-CUR/PVP-BA had a significant reduction in the number of positive *S*. Enteritidis samples in CT compared to PC.

Table 3 summarizes the effect of the therapeutic administration of 0.1% AA-CUR/PVP-BA in broiler chickens on *S*. Enteritidis colonization in the crop and CT (experiment 2). On day three post-*S*. Enteritidis challenge, no significant differences were observed in the concentration of *S*. Enteritidis in the crop and CT when comparing the PC and treated group. However, a reduction of 2.05 log and 2.71 log in *S*. Enteritidis concentration was observed in the crop and CT of treated chickens when compared with control group at 10-day post-*S*. Enteritidis challenge, respectively (Table 3). Furthermore, a significant decrease in serum FITC-d concentration and significantly lower total intestinal IgA levels were observed in broilers treated with 0.1% AA-CUR/PVP-BA when compared to PC on 10-day post-*S*. Enteritidis challenge (Table 4).

The effect of the dietary inclusion of 0.1% AA-CUR/PVP-BA on growth performance of broiler chickens in the NE model is summarized in Table 5. Seven days post *Salmonella* Typhimurium (ST) challenge, body weight (BW) and body weight gain (BWG) of the PC and AA-CUR/PVP-BA groups were significantly reduced (≈11 g in both cases) as compared to the negative control (NC) group. However, there were no significant differences in BW and BWG between the NC and AA-CUR/PVP-BA groups in the second week (7–14 d). Although PC was the group with the highest BW and BWG during the second week, the inclusion of 0.1% AA-CUR/PVP-BA into the feed resulted in a numerical increase in BWG (4.7 g) after the *Eimeria maxima* (EM) challenge (14–18 d) as compared to PC group. After the *Clostridium perfringens* (CP) challenge (day 18), 0.1% AA-CUR/PVP-BA allowed the chickens to gain 2.88 g from day 18 to day 21, meanwhile PC group reduced its BW in 11.16 g in the same period. During the last week of the trial (14–21 d), a numerical increase in BWG (≈17 g) was observed in the group supplemented with 0.1% AA-CUR/PVP-BA in comparison with the PC group. Interestingly, the feed intake (FI) accumulated (0–21 d) was significantly lower in the AA-CUR/PVP-BA group when compared to the NC and PC groups (Table 5). In the case of the feed conversion ratio (FCR), the PC group (0–21 d) had a significant and numerically lower efficiency ratio compared to the NC and AA-CUR/PVP-BA groups, respectively. Furthermore, the NC group clearly showed significant lower values in ileum lesion scores (ILS), bacterial translocation (BT) and serum FITC-d when compared to the PC or AA-CUR/PVP-BA groups (Table 6). Broilers supplemented with 0.1% AA-CUR/PVP-BA tended to have a reduction in ILS, BT and serum FITC-d concentration, when compared to the PC group (*p* = 0.07). Interestingly, the PC group showed a significant increase in total intestinal IgA levels when compared to the NC or AA-CUR/PVP-BA groups (Table 6).

## 4. Discussions

Foodborne pathogens and control of avian diseases as NE, remain high priority topics in the poultry industry [36]. However, due to the ban of antibiotic growth promoters by the spread of bacterial resistance to common antibiotics and the incidence of NE, this industry has been actively looking for other equally active molecules to avoid these problems [37,38].

In experiment 1, prophylactic administration of 0.1% AA-CUR/PVP-BA (1:1:1) in broiler chickens was capable to significantly reduce the concentration of *S*. Enteritidis in CT but not in the crop (Table 2). Previous in vitro and in vivo studies performed in our laboratory showed that 1% AA had the best antimicrobial properties against *S*. Enteritidis in the compartment that simulates the crop, but not in the intestinal compartment [19], whereas the administration of 0.1% CUR/PVP-BA into the feed significantly decreased the concentration of *S*. Enteritidis in broiler chickens [20]. Therefore, reduction in *S*. Enteritidis concentration in CT could only be related to the antimicrobial effect of CUR/PVP-BA derived from the synergistic effect between CUR/PVP and BA. 

In the second experiment, the concentration of *S*. Enteritidis in the crop and CT of broiler chickens treated with 0.1% AA-CUR/PVP-BA for 10 days post-*S*. Enteritidis challenge was significantly reduced. Probably, the decrease of *S*. Enteritidis in the crop was due to the combination of the acidifying effect of AA (0.033% in the mixture) given the release of protons in the medium (pKa = 4.1 and 11.6) [8] and the antimicrobial effect of CUR/PVP-BA (0.066% in the mixture). However, the decrease of *S*. Enteritidis in CT was closely related to the antimicrobial effect of CUR/PVP-BA since it has been reported that AA is not capable of acidifying the intestine of chicks, even when administered at 1% into the feed [39] and it is also unstable at a neutral pH [40]. The effectiveness of CUR/PVP-BA in *S*. Enteritidis reduction could be due to the improvement in the solubility and stability of CUR for its association with PVP compared to CUR alone and its interaction with BA to form complexes with better antimicrobial properties [20,41], as well as to the higher residence time in the intestine. 

Gut integrity is essential to maintain health and performance of animals [42]. *Salmonella* infections are associated with inflammation and alterations in gut permeability [43,44,45]. The results in Table 4 show that chickens treated with 0.1% AA-CUR/PVP-BA had both lower serum FITC-d concentration and total intestinal IgA levels compared to PC on day 10 post-*S*. Enteritidis challenge. FITC-d is a marker for evaluating intestinal permeability since it is a high molecular weight molecule (3–5 kDa) that is not permeable under standard conditions [32,46]. The increase in the secretion of IgA provides a critical mucosal immunity [47]. Therefore, these results confirm the decrease in the severity of *S*. Enteritidis infection given the antimicrobial activity of 0.1% AA-CUR/PVP-BA. Total intestinal IgA levels were not evaluated in experiment 1 since it has been reported that in early phases of *S*. Enteritidis infection, there are no significant differences in the secretion of intestinal IgA between infected and uninfected chickens [48,49].

Considering that the prophylactic or therapeutic administration of 0.1% AA-CUR/PVP-BA significantly reduced the concentration of *S*. Enteritidis in CT of broilers, in the third experiment the impact of the dietary administration of 0.1% AA-CUR/PVP-BA using a NE model in broiler chickens was evaluated. Many predisposing factors in NE, such as the ST and EM infection, increased the colonization and proliferation of CP, and the subsequent release of protein toxins that cause intestinal damage [25,50,51,52]. During the first seven days after the ST challenge, BW of PC and AA-CUR/PVP-BA groups was significantly reduced (*p* < 0.05) as compared to NC (Table 4), confirming that ST had a negative impact in BW [25]. However, in the second week, there were no significant differences in BW and BWG when comparing NC and AA-CUR/PVP-BA groups. Probably, the concentration of ST decreased due to the acidifying effect of AA and the antimicrobial effect of CUR/PVP-BA as described in experiment 2.

Although the *Eimeria* and CP challenges had adverse effects on performance parameters in PC group and in broiler chickens treated with 0.1% AA-CUR/PVP-BA in comparison with NC group, dietary administration of 0.1% AA-CUR/PVP-BA numerically improved FCR (0–21 d) when compared to PC group. Furthermore, broilers supplemented with 0.1% AA-CUR/PVP-BA did not show a reduction in BW after CP challenge as the PC group did, and tended to have a reduction on ILS, BT and serum FITC-d concentration, when compared to the PC group (*p* = 0.07). It has been reported that CUR has anticoccidial properties [53,54,55,56]. However, the anticoccidial mechanism of CUR has remained, but it has been proposed that it involves the induction of oxidative stress in coccidia, as well as neutralization of reactive oxygen species [2,57]. Despite that CUR/PVP was associated with BA (CUR/PVP-BA), the anticoccidial effect of CUR is not lowered since the boron molecules interact with the keto-enol groups of CUR [37,58,59] without affecting its phenolic groups, which are responsible for its anticoccidial properties [60]. Therefore, the results obtained in this experiment suggest that 0.1% AA-CUR/PVP-BA into the feed had a positive effect in slightly diminishing the effects of coccidia, a well-documented predisposing factor in NE.

Interestingly, chickens of the PC group showed a significant increase in total intestinal IgA levels when compared to NC and AA-CUR/PVP-BA groups (Table 4). Secretory IgA (SIgA) is an essential part of the adaptive humoral immune system and the primary immunoglobulin that neutralizes pathogens on external mucosal surfaces [33,61,62]. Hence, the significant decrease of IgA levels in the group supplemented with 0.1% AA-CUR/PVP-BA could be related to the anti-inflammatory properties of CUR and BA. While CUR reduces the inflammatory responses by regulating the production of some proinflammatory cytokines [56,63], BA has the ability to reduce levels of inflammatory biomarkers as TNF-α and IL-6 [64,65].

## 5. Conclusions

In conclusion, prophylactic or therapeutic administration of 0.1% AA-CUR/PVP-BA significantly reduced the concentration of *S*. Enteritidis in broiler chickens. Furthermore, dietary administration of 0.1% AA-CUR/PVP-BA had a positive effect in slightly diminishing the negative impact of NE. However, 0.1% AA-CUR/PVP-BA showed a better effect in reducing the concentration of *S*. Enteritidis when compared to the NE model. Further studies with a higher concentration of AA-CUR/PVP-BA into the feed to extend these preliminary results are currently being evaluated.

## Figures and Tables

**Table 1:** Ingredient composition and nutrient content of a basal starter diet used in the experiments on as-is basis.

Item	Starter Diet
Ingredients (g/kg)	
Corn	574.5
Soybean meal	346.6
Poultry fat ^1^	34.5
Dicalcium phosphate	18.6
Calcium carbonate	9.9
Salt	3.8
DL-Methionine	3.3
L-Lysine HCL	3.1
Threonine	1.2
Choline chloride 60%	2.0
Vitamin premix ^2^	1.0
Mineral premix ^3^	1.0
Antioxidant ^4^	0.5
Calculated analysis	
Metabolizable energy (MJ/kg)	12.7
Crude protein (g/kg)	221.5

^1^ Poultry fat West Coast Reduction LTD is primarily obtained from the tissue of poultry in the commercial process of rendering or extracting. This finished product was used as an energy source for animal and aquaculture feed. ^2^ Vitamin premix supplied per kg of diet: Retinol, 6 mg; cholecalciferol, 150 µg; dl-α-tocopherol, 67.5 mg; menadione, 9 mg; thiamine, 3 mg; riboflavin, 12 mg; pantothenic acid, 18 mg; niacin, 60 mg; pyridoxine, 5 mg; folic acid, 2 mg; biotin, 0.3 mg; cyanocobalamin, 0.4 mg. ^3^ Mineral premix supplied per kg of diet: Mn, 120 mg; Zn, 100 mg; Fe, 120 mg; copper, 10 mg to 15 mg; iodine, 0.7 mg; selenium, 0.2 mg; and cobalt, 0.2 mg. ^4^ Ethoxyquin.

**Table 2:** Prophylactic administration of a 0.1% mixture containing ascorbic acid (AA) and a solid dispersion of curcumin with polyvinylpyrrolidone (CUR/PVP, ratio 1:9) and boric acid (BA) (AA-CUR/PVP-BA) on crop and cecal tonsils (CT) colonization of *Salmonella* Enteritidis (*S*. Enteritidis) ^1^ in broiler chickens (Experiment 1).

Treatments	Crop *S*. Enteritidis Log cfu/g	Crop *S*. Enteritidis Incidence	CT *S*. Enteritidis Log cfu/g	CT *S*. Enteritidis Incidence
Positive control AA-CUR/PVP-BA	Trial 1			
	2.68 ± 0.47 ^a^	9/12 (75%)	4.01 ± 0.29 ^a^	12/12 (100 %)
	2.60 ± 0.45 ^a^	10/12 (83%)	2.32 ± 0.50 ^b^	8/12 (67 %) *
Positive control AA-CUR/PVP-BA	Trial 2			
	2.69 ± 0.48 ^a^	9/12 (75%)	3.94 ± 0.22 ^a^	12/12 (100 %)
	2.57 ± 0.55 ^a^	7/12 (58%)	2.28 ± 0.59 ^b^	7/12 (58 %) **

Data are presented in Log cfu/g of tissue. Mean ± SE from 12 chickens. ^1^ Chickens were orally gavaged with 10^7^ cfu of *S*. Enteritidis per chicken at six-day old, samples were collected 24 h later. ^a,b^ Values within treatment columns for each treatment with different superscripts differ significantly (*p* < 0.05). For *S*. Enteritidis incidence, data are presented as positive/total chickens (percentage). * *p* < 0.05; ** *p* < 0.01.

**Table 3:** Therapeutic administration of a 0.1% mixture containing ascorbic acid (AA) and a solid dispersion of curcumin with polyvinylpyrrolidone (CUR/PVP, ratio 1:9) and boric acid (BA) (AA-CUR/PVP-BA) on crop and cecal tonsils (CT) colonization of *Salmonella* Enteritidis (*S*. Enteritidis) ^1^ in broiler chickens at three and ten days post-*S*. Enteritidis challenge (experiment 2).

Treatments	Crop *S*. Enteritidis Log cfu/g	Crop *S*. Enteritidis Incidence	CT *S*. Enteritidis Log cfu/g	CT *S*. Enteritidis Incidence
Positive control AA-CUR/PVP-BA	Three days post-*S*. Enteritidis challenge			
	3.18 ± 0.46 ^a^	10/12 (83%)	6.44 ± 0.15 ^a^	12/12 (100%)
	2.21 ± 0.48 ^a^	8/12 (67%)	5.33 ± 0.73 ^a^	10/12 (83%)
Positive control AA-CUR/PVP-BA	Ten days post-*S*. Enteritidis challenge			
	2.93 ± 0.65 ^a^	7/12 (58%)	6.61 ± 0.21 ^a^	12/12 (100%)
	0.88 ± 0.46 ^b^	3/12 (25%)	3.90 ± 0.86 ^b^	8/12 (67%) *

Data are presented in Log cfu/g of tissue. Mean ± SE from 12 chickens. ^1^ Chickens were orally gavaged with 10^4^ cfu of *S*. Enteritidis per chicken at 1-day old, samples were collected three and ten days post-*S*. Enteritidis challenge. ^a,b^ Values within treatment columns for each treatment with different superscripts differ significantly (*p* < 0.05). For *S*. Enteritidis incidence, data are presented as positive/total chickens (percentage). * *p* < 0.05.

**Table 4:** Therapeutic administration of a 0.1% mixture containing ascorbic acid (AA) and a solid dispersion of curcumin with polyvinylpyrrolidone (CUR/PVP, ratio 1:9) and boric acid (BA) (AA-CUR/PVP-BA), on serum concentration of fluorescein isothiocyanate-dextran (FITC-d), and total intestinal immunoglobulin A (IgA) levels in broiler chickens on day ten post *Salmonella* Enteritidis (*S*. Enteritidis) challenge ^1^ (experiment 2).

Treatments	FITC-d (μg/mL)	IgA (μg/mL)
Positive control	0.700 ± 0.020 ^a^	14.34 ± 2.81 ^a^
AA-BA-CUR/PVP	0.489 ± 0.026 ^b^	7.38 ± 1.08 ^b^

Data expressed as mean ± SE from 12 chickens. ^1^ Chickens were orally gavaged with 10^4^ cfu of *S*. Enteritidis per chicken at 1-d old, samples were collected ten days post-*S*. Enteritidischallenge. ^a,b^ Values within columns with different superscripts differ significantly (*p* < 0.05).

**Table 5:** Evaluation of body weight (BW), body weight gain (BWG), feed intake (FI) and feed conversion ratio (FCR) in broiler chickens consuming a diet supplemented with or without a 0.1% mixture containing ascorbic acid (AA) and a solid dispersion of curcumin with polyvinylpyrrolidone (CUR/PVP, ratio 1:9) and boric acid (BA) (AA-CUR/PVP-BA) on a Necrotic enteritis challenge model ^1^ (Experiment 3).

Item	Negative Control	Positive Control	AA-CUR/PVP-BA
BW, g/broiler			
d 0	46.88 ± 0.64 ^a^	46.54 ± 0.64 ^a^	47.24 ± 0.66 ^a^
d 7	127.14 ± 2.90 ^a^	115.58 ± 3.27 ^b^	115.69 ± 3.15 ^b^
d 14	273.80 ± 11.02 ^a^	295.78 ± 12.10 ^a^	264.60 ± 10.91 ^a^
d 18	457.79 ± 18.97 ^a^	456.32 ± 19.39 ^a^	436.14 ± 16.41 ^a^
d 21	603.81 ± 24.32 ^a^	445.16 ± 18.50 ^b^	438.91 ± 17.79 ^b^
BWG, g/broiler			
d 0–7	80.39 ± 3.06 ^a^	67.74 ± 3.24 ^b^	68.46 ± 3.18 ^b^
d 7–14	147.01 ± 9.51 ^b^	182.60 ± 9.48 ^a^	149.89 ± 8.83 ^b^
d 14–18	183.99 ± 9.85 ^a^	160.55 ± 9.02 ^a^	165.25 ± 6.72 ^a^
d 14–21	325.78 ± 15.58 ^a^	152.13 ± 9.67 ^b^	169.11 ± 9.78 ^b^
d 0–21	552.72 ± 24.35 ^a^	399.42 ± 19.79 ^b^	395.12 ± 17.46 ^b^
FI, g/broiler			
d 0–21	808.21 ± 29.86 ^a^	772.34 ± 10.66 ^a^	685.05 ± 25.21 ^b^
FCR			
d 0–21	1.46 ± 0.04 ^b^	1.93 ± 0.10 ^a^	1.73 ± 0.15 ^a^

^1^ Data expressed as mean ± SE from 40 chickens. ^a,b^ Values within columns with different superscripts differ significantly (*p* < 0.05).

**Table 6:** Evaluation of a 0.1% mixture containing ascorbic acid (AA) and a solid dispersion of curcumin with polyvinylpyrrolidone (CUR/PVP, ratio 1:9) and boric acid (BA) (AA-CUR/PVP-BA) on ileum NE lesion scores (ILS), bacterial translocation (BT) to the liver, serum concentration of fluorescein isothiocyanate–dextran (FITC-d) and immunoglobulin A (IgA) levels in broiler chickens ^1^.

Treatments	ILS ^2^	BT Log_10_ cfu/g ^3^	FITC-d (μg/mL) ^4^	IgA (μg/mL) ^5^
Negative Control	0.33 ± 0.12 ^b^	1.52 ± 0.46 ^b^	0.312 ± 0.048 ^b^	36.14 ± 3.79 ^b^
Positive Control	2.04 ± 0.18 ^a^	3.34 ± 0.46 ^a^	0.692 ± 0.050 ^a^	50.85 ± 4.48 ^a^
AA-CUR/PVP-BA	1.92 ± 0.13 ^a^	3.09 ± 0.54 ^a^	0.553 ± 0.056 ^a^	35.35 ± 2.07 ^b^

^1^ Data expressed as mean ± SE. ^2^ ILS was evaluated in 25 broiler chickens. ^3^ BT was expressed in Log_10_ cfu /g of tissue from 12 chickens. ^4^ FITC-d concentration of 20 serum samples. ^5^ IgA levels determined in 12 intestine samples. ^a,b^ Values within treatment columns for each treatment with different superscripts differ significantly (*p* < 0.05).
